# Teletherapie nach Cochleaimplantation in der COVID-19-Pandemie

**DOI:** 10.1007/s00106-021-01124-y

**Published:** 2021-11-26

**Authors:** Christiane Völter, Carolin Stöckmann, Hannah Klein, Stefan Dazert, Jan Peter Thomas

**Affiliations:** 1 Klinik für Hals-Nasen-Ohrenheilkunde, Kopf- und Halschirurgie, Ruhr-Universität Bochum, St. Elisabeth-Hospital, Bleichstraße 15, 44787 Bochum, Deutschland; 2 Klinik für Hals-Nasen-Ohrenheilkunde, Kopf- und Halschirurgie, Kath. St. Paulus Gesellschaft, St. Johannes Hospital Dortmund, Johannesstr. 9–17, 44137 Dortmund, Deutschland

**Keywords:** Hörrehabilitation, Videotherapie, Usability, Therapeutische Allianz, Cochleaimplantation, Auditory rehabilitation, Video therapy, Usability, Therapeutic alliance, Cochlear implantation

## Abstract

**Hintergrund:**

Die Digitalisierung im Gesundheitswesen hat unter der COVID-19-Pandemie rasant zugenommen. Bislang fand Hörtraining nach Cochleaimplantation meist Face-to-Face statt, doch die Kontaktvermeidung erschwert diesen Therapieansatz.

**Material und Methoden:**

Insgesamt 42 erwachsene Cochleaimplantat(CI)-Träger im Alter von 53,8 (±15,6) erhielten 1×/Woche über 5 Wochen Videotherapie im Rahmen der Folgetherapie nach Cochleaimplantation. Nach jeder Therapieeinheit erfolgte eine Dokumentation hinsichtlich des Ablaufs und der Therapieinhalte. Nach Studienende wurden neben einer Kosten-Nutzen-Analyse das Konzept und die Benutzerfreundlichkeit anhand der System Usability Scale (SUS) und eines eigenen Abschlussfragebogens zur Videotherapie sowie die Therapeuten-Patienten-Beziehung mit der Skala Therapeutische Allianz – Revised (STA-R) sowohl von Patienten als auch von Therapeuten bewertet.

**Ergebnisse:**

Gleichermaßen hoch schätzten beide Usergruppen die Benutzerfreundlichkeit ein (87,9 vs. 93,0). Trotz des fehlenden persönlichen Kontakts wurde die therapeutische Allianz sehr positiv angesehen (87,8 % vs. 84,8 %). Die therapeutischen Bedürfnisse der Patienten konnten in 47,6 % vollständig durch die Videotherapie abgedeckt werden. Der größte Vorteil für die Patienten lag in der Zeit- und Kostenersparnis. Für die Rehabilitationseinrichtung entstanden zunächst mehr Kosten aufgrund einer längeren Therapievorbereitung. Auch traten in > 75 % der ersten Therapieeinheiten technische Probleme auf. Langfristig war die Durchführung der Therapie hierdurch nicht beeinträchtigt.

**Schlussfolgerung:**

Videogestütztes Hörtraining wird als nützlich beurteilt und auch zukünftig gewünscht. Ob die positiv erlebte therapeutische Allianz auch über einen längeren Therapiezeitraum aufrechtzuerhalten sein wird und wie effektiv Videotherapie ist, bedarf weiterer Studien.

## Abstract (en)

**Background:**

Due to the COVID-19 pandemic, digitalization in healthcare grew rapidly. Auditory training after cochlear implantation usually takes place face-to-face but social distancing interferes with this therapeutic approach.

**Materials and Methods:**

In follow-up treatment, 42 adult cochlear implant (CI) users aged 53.8 (±15.6) years received video therapy 1 x/week for 5 weeks on a certified platform. After each therapy session, the technical process and therapeutic content were assessed. At the end of the study, usability and the relationship between therapist and patient were evaluated by patients and therapists using the System Usability Scale (SUS), a final questionnaire and by the Skala Therapeutische Allianz – Revised (STA-R). Furthermore, a cost–benefit analysis was done.

**Results:**

Usability for both users was high (87.97 versus 93.0). Despite the lack of personal contact, therapeutic alliance was highly appreciated by patients and therapists (87.8% versus 84.8%). The main advantages for the patients were reductions in time and costs. In contrast, the rehabilitation center faced higher costs initially due to the longer time therapists needed to prepare the lessons. Technical problems had to be solved in > 75% of the first sessions but did not bother training thereafter. In total, 47.6% of the patients believe that teletherapy can completely fulfill their therapeutic needs.

**Conclusion:**

Video therapy has been judged as a useful tool by all users and the majority wants to continue. However, it remains questionable whether the therapist–patient relationship can be sufficiently maintained over a longer period and whether online therapy is as effective as face-to-face therapy.

## Hintergrund

Auch wenn in den vergangenen Jahren eine rasante technologische Entwicklung zu verzeichnen ist und digitale Medien bereits fester Bestandteil unseres Alltags geworden sind, fanden diese in Deutschland bis vor Kurzem nur schleppend Einzug in die klinische Routine. Die COVID-19-Pandemie hat diese Situation grundlegend geändert [19]. Über Nacht mussten digitale Lösungen gefunden und implementiert werden, um die medizinische und therapeutische Versorgung der Patienten zu gewährleisten [13, 17, 20]. Dies trifft auch auf die Hörrehabilitation nach einer Cochleaimplantation zu [3, 4].

Basierend auf der Sk2-Leitlinie der Deutschen Gesellschaft für Hals-Nasen-Ohren-Heilkunde, Kopf- und Hals-Chirurgie gliedert sich der postoperative Rehabilitationsprozess nach einer Cochleaimplantation in drei Phasen [14]. Im Rahmen der Basistherapie, beginnend zwischen dem ersten postoperativen Tag und bis 6 Wochen postoperativ, werden unter stationären Bedingungen oder bei günstigen Voraussetzungen und enger Strukturierung unter ambulanten Bedingungen u. a. die Ersteinstellung und Optimierung des Sprachprozessors, die Initiierung der Hör-Sprach-Therapie sowie technische und audiometrische Kontrollen durchgeführt, während in der dann bei erwachsenen CI-Trägern anschließenden 6‑ bis 24-monatigen Folgetherapie die auditiven Fähigkeiten weiter ausgebaut werden. Hieran schließt sich die lebenslange Nachsorge an mit dem Ziel, eine kontinuierliche medizinische, therapeutische und technische Versorgung der Patienten zu gewährleisten. Dem Hörtraining selbst liegt eine 4‑stufige Struktur zugrunde, welche systematisch durchlaufen wird, beginnend mit der Wahrnehmung (Detektion) auditiver Reize, der Analyse ihrer Eigenschaften (Länge, Lautstärke, Tonhöhe), dem Unterscheiden (Differenzierung) und Erkennen (Identifikation) derselben. Hilfen, wie das Schriftbild, semantische Einschränkungen oder das Mundbild werden sukzessive abgebaut, bis Patienten Sprache ohne Mundbild im Störgeräusch verstehen können.

Bislang fand die postoperative Rehabilitation im Rahmen der Folgetherapie nach einer Cochleaimplantation in Deutschland meist Face-to-Face in speziellen Rehabilitationseinrichtungen ambulant oder stationär statt [14, 24, 38, 44, 48]. Im Zuge der Digitalisierung finden zunehmend auch Onlineangebote Einzug in den CI-Rehabilitationsprozess. Aktuell handelt es sich hierbei meist um Applikationen, die Patienten additiv zur konventionellen Therapie nutzen. Diesen mangelt es häufig an Komplexität, Adaptivität, Feedback- und Motivationselementen [43]. Computerbasierte Trainingsplattformen, die eine standardisierte Online-Hörrehabilitation in Analogie zum Training vor Ort beinhalten, sind noch in der Entwicklung [45]. Videotherapeutische Settings wurden bisher in der Logopädie vornehmlich zur Behandlung sprachtherapeutischer Störungsbilder, wie der Aphasie, Dysphagie, Dysphonie und des Stotterns bei Patienten unterschiedlichen Alters eingesetzt [6, 16, 22, 29, 33, 34, 46].

Demgegenüber erfolgt in der Psychotherapie seit Jahren eine digitale Betreuung von Patienten. Diese wird nicht nur von den Patienten selbst als positiv wahrgenommen [32], sondern ihre Effektivität konnte auch bereits in verschiedenen Studien belegt werden [13, 27, 35].

Ein wichtiger Einwand gegen jede Form der Videotherapie ist die Befürchtung aufseiten der Therapeuten, dass sich durch videotherapeutische Interventionen die Beziehung zwischen Patient und Therapeut negativ verändere und es ohne einen persönlichen Kontakt schwieriger sei, eine therapeutische Allianz aufzubauen oder diese aufrechtzuerhalten [17]. Das Konzept der therapeutischen Allianz, das nicht nur das emotionale Band zwischen Patient und Therapeut, sondern auch die Übereinstimmung im Hinblick auf die Ziele und die Art der therapeutischen Übungen umfasst, wird jedoch als einer der Schlüsselfaktoren für den Erfolg einer Therapie angesehen [10, 36]. Im Hinblick auf Videotherapien wurde dieser Aspekt bislang nicht ausreichend untersucht [7, 8, 40].

Ziel der vorliegenden Studie war es, den Einsatz der Videotherapie im Rahmen der postoperativen Rehabilitation bei erwachsenen CI-Trägern aus Sicht der Therapeuten und der Patienten im Hinblick auf Nutzerfreundlichkeit, die therapeutische Allianz sowie die technische und inhaltliche Umsetzung zu evaluieren.

## Material und Methoden

### Teilnehmer

In die Studie wurden 42 erwachsene CI-Träger, die sich in der Folgetherapie nach Cochleaimplantation befanden, mit einem Durchschnittsalter von 58,8 (±15,4) Jahren, einer Hörerfahrung von mindestens zwei Monaten (Ø 11,2; ±7,1) und einem Einsilberverstehen von 51 % (±25 %) gemessen am Freiburger bei 65 dB eingeschlossen (Tab. 1). Bei 33 CI-Trägern lag eine bilaterale (hiervon in 6 Fällen eine bilaterale CI-, in 27 Fällen eine bimodale Versorgung), bei 9 eine einseitige Hörstörung (Single-Sided Deafness, SSD) vor. Als Einschlusskriterien galten: (1) Alter ≥ 18 Jahre, (2) keine schwere motorische, visuelle oder kognitive Beeinträchtigung, (3) Vorhandensein einer entsprechenden technischen Ausstattung beim Patienten (Tab. 1). Daneben nahmen 5 Logopäden im Alter von 27–59 Jahren mit Erfahrung in der Hörrehabilitation teil.

**Table 1:** 

Alter (Jahre)	Ø 53,8 [15,4]
Geschlecht	27 Frauen; 15 Männer
Bildungsjahre^a^	Ø 11,35 [2,16]
Art der Versorgung	6 bilaterales CI
	27 bimodale Versorgung
	9 unilaterales CI
Dauer der Hörstörung	Ø 18,17 [14,41]
Hörerfahrung mit dem CI (Monate)	Ø 11,2 [7,1]
Grad der Hörstörung auf Gegenohr (4-PTA)	Ø 59,5 [39,75]
Anreise (km)	Ø 79,5 [60,6]
Anreisedauer (min)	Ø 107,4 [53,8]

^a^Definiert nach [49]

### Videotherapie

Die während der COVID-19-Pandemie kostenlos zur Verfügung stehende Plattform sprechstunde.online® (https://sprechstunde.online/) ist nach Anlage 31b zur IT-Sicherheit und zum Datenschutz zertifiziert und gewährleistet eine End-zu-End-Verschlüsselung über die gesamte Therapielaufzeit [26]. Nach der Registrierung erhalten die Therapeuten einen persönlichen Account, die Patienten dagegen erhalten über einen einmaligen, individuellen Sicherheitscode Zugriff auf die Plattform. Dieser wird nach der Terminerstellung durch den Therapeuten automatisch generiert und dem Patienten via E‑Mail oder SMS zur Verfügung gestellt.

Im Rahmen der Videotherapie können Beratungs- und Übungssequenzen durchgeführt werden. Neben einer simultanen Bild- und Tonübertragung erlaubt das Programm auch ein Teilen des Bildschirms sowie das Senden und Empfangen von pdf-Dokumenten und den Austausch schriftlicher Informationen per Chat. Im Rahmen der vorliegenden Studie arbeiteten Therapeuten an feststehenden Computern mit einer externen Webcam der Fa. Logitech (Apples, Schweiz) (Modell C270), die die Bild- und Tonübertragung gewährleistete. Um möglichst vielen Patienten die Teilnahme am Videotherapieangebot zu ermöglichen, wurde das Endgerät nicht eingegrenzt. Die Nutzung von Smartphone, Tablet, Laptop und Computern war möglich. Eine Verbindung über eine Audioschleife wurde empfohlen.

### Studienaufbau

Alle Therapeuten erhielten eine interne Einweisung. Registrierungen, Terminvergabe, Equipment und mögliche Störungsgründe wurden thematisiert. Im Rahmen von internen Testdurchläufen wurde der Umgang gefestigt. Basierend auf den Erfahrungen wurde eine Bedienungsanleitung für die Patienten erstellt. Telefonisch standen Therapeuten für Rückfragen zur Verfügung. Alle Patienten erhielten einmal wöchentlich eine Videotherapie à 45 min als Einzeltraining über 5 Wochen. Nach jeder Therapieeinheit bewerteten die Therapeuten die Sitzung mittels eines eigens hierfür entwickelten Kurzfragebogens zur Videotherapie (Tab. 2). Daneben wurde nach Studienende die Anwendbarkeit des Programms, die Therapeuten-Patienten-Beziehung und die Qualität des Trainings mithilfe folgender Fragebögen sowohl durch den Patienten als auch den Therapeuten erfasst.

**Table 2:** 

Qualität der Therapieeinheit	Die allgemeine Qualität dieser Teletherapie war sehr gut
	Die Tonqualität dieser Teletherapie war sehr gut
	Die Bildqualität dieser Teletherapie war sehr gut
	Ich habe mich während der Teletherapie wohl gefühlt
	Es gab technische Probleme, die die Qualität der Therapie beeinflusst haben
	*Wenn ja, welche?*
	Es gab organisatorische Probleme, die die Qualität der Therapie beeinflusst haben
	Der Patient brauchte/die Eltern des Patienten brauchten bei der Durchführung Videokonferenz Unterstützung
	*Wenn ja, beschreiben Sie bitte das Problem*
	Ich selbst brauchte Hilfe bei der Durchführung der Videokonferenz
	*Wenn ja, beschreiben Sie bitte das Problem*
Therapieinhalte	Ich konnte die geplanten Therapieinhalte umsetzen
	Die Vorbereitung für die Videokonferenz ist anders als die Vorbereitung der Vor-Ort-Therapie
	*Wenn ja, wie?*
	Ich habe Übungen zu folgenden Bereichen durchgeführt (Mehrfachnennungen möglich). □ Geräusche □ Laute □ Silben □ Wörter □ Sätze □ Texte □ Elternberatung □ Wortschatz □ Grammatik □ Aussprache □ Merkfähigkeit Andere:_________________________________________________________
Sonstiges	*Die wievielte Videokonferenz war es für den Patienten?*
	*Welches Endgerät hat der Patient genutzt?*
	*Welche zusätzlichen Hilfsmittel hat der Patient genutzt (Audiolink, Bluetooth-Schleife etc.)?*

#### System Usability Scale (SUS)

Die Nutzerfreundlichkeit wurde anhand der SUS erfasst und entsprechend der Arbeit von Lewis (2018) [31] ausgewertet. Dabei werden die Rohwerte basierend auf einer Likert-Skala von 0–4 entsprechend der Konnotation der Fragestellung umgewandelt. Dabei entsprechen 4 Punkte der bestmöglichen und 0 Punkte der schlechtesten Bewertung. Die umgewandelten Werte werden mit dem Faktor 2,5 multipliziert, um einen Maximalscore von 100 zu erhalten. Des Weiteren werden die Einzelfragen separat beurteilt. Anhand der in Tab. 3 dargestellten Werte wird deutlich, wie hoch die prozentuale Bewertung der Fragen im Vergleich mit deren Maximalwert ist.

**Table 3:** 

		Patienten	Therapeuten
		% (SD)	% (SD)
1	Ich kann mir gut vorstellen, das Programm regelmäßig zu nutzen	83,9 (25,79)	95,0 (11,18)
2	Ich empfinde das Programm als einfach zu nutzen	91,7 (18,03)	95,0 (11,18)
3	Ich finde, dass die verschiedenen Funktionen des Programms gut integriert sind	86,3 (24,82)	80,0 (27,39)
4	Ich kann mir vorstellen, dass die meisten Leute die Bedienung schnell erlernen	81,0 (23,95)	70,0 (20,92)
5	Ich habe mich bei der Nutzung des Programms sicher gefühlt	90,5 (14,56)	95,0 (11,18)
6	Ich empfinde das Programm als unnötig komplex	95,2 (17,67)	100,0 (0,00)
7	Ich denke, dass ich technische Unterstützung brauchen würde, um die Videotherapie zu nutzen	83,3 (29,56)	100,0 (0,00)
8	Ich finde, dass es in dem Programm zu viele Widersprüche gibt	93,5 (29,56)	100,0 (0,00)
9	Ich empfinde die Bedienung als sehr umständlich	89,3 (25,39)	95,0 (11,18)
10	Ich musste eine Menge lernen, bevor ich mit dem Programm arbeiten konnte	85,1 (27,08)	100,0 (0,00)

#### Skala Therapeutische Allianz – Revised (STA-R)

Die Therapeuten-Patienten-Beziehung wurde mit dem STA-R-Fragebogen [10] auf einer Likert-Skala von 0–4 (von „Ich stimme gar nicht zu“ bis „Ich stimme voll zu“) evaluiert. Hierin finden sich 17 Items auf 4 Skalen zur zuversichtlichen Zusammenarbeit mit dem Therapeuten (1), der positiven emotionalen Beziehung zum Therapeuten (2) und Fragen, wie gut es dem Patienten in der Videotherapie gelingt, sich zu öffnen (3), aber auch Fragen nach einer möglichen Interferenz mit der Therapeutenpersönlichkeit (4).

#### Abschlussfragebogen zur Videotherapie

Der Fragebogen für Patienten (Tab. 4) umfasste 6, der für Therapeuten (Tab. 5) 9 verschiedene Aspekte. Mit Ausnahme der Fragen 42 und 43 des Therapeutenfragebogens wurden alle Items in Bezug auf jeden einzelnen Teilnehmer auf einer Likert-Skala von 0–4 bewertet. Höhere Punktzahlen verweisen auf bessere Ergebnisse. Um die Bewertung der Einzelfragen darzustellen, wurden Prozentwerte gewählt. Diese zeigen auf, wieviel Prozent der Maximalpunktzahl der entsprechenden Frage erreicht wurde. Zusätzlich wurden ökonomische Daten auf Basis der Entfernung zum CI-Zentrum und der Zeit, um CI-Zentrum zu erreichen, und Daten zur technischen Vorerfahrung der Patienten erhoben.

**Table 4:** 

		% (SD)
Nutzen	1 Telemedizin verbessert meinen Zugang zu Gesundheitsdiensten	80,4 (25,02)
	2 Telemedizin erspart mir die Zeit für die Anreise zum CI-Zentrum	96,4 (10,44)
	3 Telemedizin deckt meinen Bedarf an therapeutischer Versorgung ab	61,9 (27,18)
Nutzerfreundlichkeit und Erlernbarkeit	4 Es war einfach, das System zu benutzen	88,7 (18,48)
	5 Der Umgang mit dem System war leicht zu erlernen	91,1 (20,53)
	6 Mit diesem System könnte ich meines Erachtens schnell effektiv arbeiten	88,1 (21,55)
Qualität des Programms	7 Der Umgang mit diesem System war angenehm	89,3 (19,24)
	8 Ich habe das System gerne benutzt	87,5 (22,93)
	9 Das System war einfach und leicht verständlich	89,3 (19,24)
	10 Das System konnte alles, was ich mir gewünscht habe	82,7 (26,76)
Qualität der Interaktion	11 Ich konnte mich mit der Therapeutin leicht unterhalten	88,1 (21,55)
	12 Ich konnte die Therapeutin gut verstehen	86,9 (20,84)
	13 Ich hatte das Gefühl, mich adäquat ausdrücken zu können	90,5 (23,40)
	14 Ich konnte die Therapeutin während der Videokonferenz gut sehen	80,4 (21,76)
Zufriedenheit und zukünftiger Einsatz	15 Ich habe ein gutes Gefühl bei der Videokonferenzen gehabt	89,9 (19,95)
	16 Ich denke, die Therapie über die Videokonferenzen ist gleichermaßen effektiv wie eine Face-to-Face-Therapie	69,6 (31,98)
	17 Videokonferenzen sind ein gutes Mittel, um Therapien durchzuführen	85,1 (22,13)
	18 Ich würde die Videokonferenz gerne weiterhin nutzen	79,8 (29,34)
	19 Insgesamt bin ich mit der Videotherapie zufrieden	89,3 (19,24)
	20 Die Videokonferenz ist eine Alternative zum Hörtraining vor Ort	79,2 (31,19)
	21 Die Videokonferenz ist eine Bereicherung für das Hörtraining	89,3 (17,59)
	22 Ich habe mich auf die Videokonferenzen gefreut	86,3 (24,20)
Therapieinhalte	23 Der Inhalt der Videokonferenzen ähnelte den Therapien vor Ort	88,7 (20,81)
	24 Die Therapie über die Videokonferenz war abwechslungsreich	92,3 (15,11)
	25 Die Therapieinhalte waren individuell auf mich abgestimmt	95,8 (10,93)

**Table 5:** 

		% (SD)
Nutzen	1) Telemedizin kann den therapeutischen Bedarf meines Patienten vollständig abdecken	64,9 (19,95)
Nutzerfreundlichkeit und Erlernbarkeit	2) Es war einfach, das System zu benutzen	95,8 (10,93)
	3) Der Umgang mit dem System war leicht zu erlernen	96,4 (10,44)
	4) Mit diesem System könnte ich meines Erachtens schnell effektiv arbeiten	91,1 (13,32)
Qualität des Programms	5) Der Umgang mit diesem System war angenehm	88,7 (17,64)
	6) Ich habe das System gerne benutzt	81,5 (20,70)
	7) Das System war einfach und leicht verständlich	98,8 (5,39)
	8) Das System enthielt alles, was ich mir gewünscht habe	73,2 (30,94)
Qualität der Interaktion	9) Ich konnte mich leicht mit meinem Patienten unterhalten	89,3 (18,43)
	10) Ich konnte den Patienten gut verstehen	89,9 (14,67)
	11) Ich konnte den Patienten während der Videokonferenzen gut sehen	82,7 (19,51)
	12) Der Patient hat sich während der Videotherapie ähnlich verhalten wie bei dem Vor-Ort-Training	94,6 (17,92)
Technische Probleme	13) Wann immer ich bei der Benutzung des Systems einen Fehler gemacht habe, konnte ich ihn leicht und schnell beheben	88,7 (18,48)
Zufriedenheit und zukünftiger Einsatz	15) Insgesamt bin ich mit der Videotherapie zufrieden	86,9 (16,78)
	16) Ich hatte ein gutes Gefühl bei der Verwendung der Videokonferenz	89,9 (13,59)
	17) Die Videokonferenz ist eine Alternative zum Hörtraining vor Ort	79,2 (20,60)
	18) Die Videokonferenz ist eine Bereicherung für das Hörtraining	91,7 (11,93)
	19) Ich würde die Videokonferenz gerne weiterhin nutzen	82,1 (18,55)
	20) Die Durchführung der Teletherapie ist für mich genauso erfüllend wie die Vor-Ort-Therapie	50,6 (23,09)
	21) Die Videokonferenz kann die Therapie vor Ort ersetzen	58,9 (18,98)
Therapievorbereitung	22) Ich konnte vorhandenes Therapiematerial für die Videokonferenz nutzen	88,7 (21,53)
	23) Die Therapievorbereitung hat mich mehr Zeit gekostet als sonst	58,9 (18,98)
Therapieinhalte	28) Übungen zu Geräuschen konnte ich problemlos integrieren	Auswertung lässt keine prozentualen Aussagen zu
	29) Übungen zu Silben konnte ich problemlos integrieren	
	30) Übungen zum Wortverständnis konnte ich problemlos integrieren	
	31) Übungen zum Satzverständnis konnte ich problemlos integrieren	
	32) Übungen zum Textverständnis konnte ich problemlos integrieren	
	33) Übungen im Live Voice konnte ich problemlos integrieren	
	34) Computerbasierte Aufgaben (Audiolog, Tonaufnahmen) konnte ich problemlos integrieren	
	35) Spielerische Inhalte konnte ich problemlos integrieren	
	36) Das Schriftbild konnte ich problemlos als Hilfestellung nutzen	
	37) Das Mundbild konnte ich problemlos als Hilfestellung nutzen	
Persönliche Einstellung	42) Bereits vor der Studie habe ich Teletherapie als Chance für die Versorgung von CI-Patienten gesehen	65,0 (28,50)
	43) Meine Einstellung zu Teletherapien hat sich durch die Studie geändert	50,0 (50,00)

#### Kurzfragebogen zur Videotherapie

 Der eigens für die Studie entwickelte Kurzfragebogen für Therapeuten umfasste die Bereiche Beurteilung der Qualität und Therapieinhalte (Tab. 2). Die 10 Auswahlfragen wurden in Bezug auf jeden einzelnen Teilnehmer auf einer Likert-Skala von 0–4 bewertet. Eine höhere Punktzahl in Bezug auf technische Fragen bedeutete ein besseres, in Bezug auf das Therapiematerial und die therapeutische Umsetzung ein schlechteres Ergebnis. Außerdem beinhaltete der Fragebogen vier offene Fragen, die der Spezifizierung der Rückmeldung dienen. Der Fragebogen wurde nach jeder Therapieeinheit (TE) (1–5) ausgefüllt.

#### Trainingsmaterial

Gearbeitet wurde vorrangig Screen-to-Screen mit im Live-Voice-Modus präsentierten Inhalten, wobei die Lautstärke entsprechend dem Pegel eingestellt wurde, der für den Patienten angenehm war. Abhängig vom Leistungsstand der Patienten wurden Übungen angepasst an die individuellen Voraussetzungen und das Lerntempo ausgewählt [47]. Übergeordnetes Ziel war es, phonetische, semantische und visuelle Hilfen schrittweise abzubauen, um ein freies Sprachverstehen zu erzielen. Dazu wurden zunächst die Geräuschwahrnehmung und -diskrimination sowie die Wahrnehmung von rhythmisch-prosodischen Sprachstrukturen (Wort- und Satzlänge) trainiert. Es folgten Vokal- und Konsonantenunterscheidung sowie Identifikationsaufgaben in Bezug auf Zahlen, Wörter und Sätze im Closed und Open Set, zunächst in Ruhe, später im Störgeräusch.

#### Ökonomische Analyse

Um eine Gegenüberstellung der Kosten seitens der Patienten und der Therapeuten zu ermöglichen, wurden die Kosten der Patienten basierend auf den Reisekosten ermittelt. Pro gefahrenen Kilometer für die Anreise zum CI-Zentrum wurde die aktuell gültige Kilometerpauschale von 0,30 € veranschlagt. Die Kosten seitens der Therapeuten wurden basierend auf der investierten Arbeitszeit berechnet, wobei ein Stundenlohn von 35 €, basierend auf der aktuellen Vergütungssituation in der Einrichtung, angesetzt wurde. Kosten für die Nutzung des Internets, Raummiete oder Neuanschaffungskosten für technisches Equipment wurden nicht berücksichtigt.

### Statistische Auswertung

Zunächst wurde eine deskriptive Analyse der Daten mithilfe des Mittelwerts und der Standardabweichung durchgeführt. Korrelationen zwischen den Aussagen und den soziodemografischen Faktoren wie Alter und Geschlecht wurden berechnet. Falls keine Normalverteilung vorlag, wurde die Kendall-Rangkorrelation bei Rangbindung angewendet. Der Vergleich zwischen den Therapeuten- und Patientendaten wurde mit dem Mann-Whitney-U-Test vorgenommen. Das Signifikanzniveau wurde auf *p* = 0,05 festgelegt. Die statistische Analyse erfolgte mit Medas (Fa. Grund, Margetshöchheim).

Ein positives Ethikvotum (Nr. 20-6936) der Ethikkommission der Medizinischen Fakultät der Ruhr-Universität Bochum lag vor.

## Ergebnisse

### System Usability Scale (SUS)

Mit einem Durchschnittswert von 93,0 (±3,6) bewerteten die Therapeuten die Benutzerfreundlichkeit des Programms als ausgezeichnet. Die Bewertung der Patienten fiel etwas geringer aus (87,9 ±13,85), unterschied sich jedoch nicht signifikant (*p* = 0,67). Eine Analyse der einzelnen Fragen (Tab. 3) zeigt, dass sich sowohl Patienten (90,5 % ±14,56) als auch Therapeuten (95,0 % ±11,18) im Umgang mit dem Programm sicher fühlten und die Bedienung des Programms als einfach einschätzten (Patienten: 91,7 %  ±18,03; Therapeuten: 95,0 % ±11,18). Kritisch wurde die Frage nach einem selbstständigen Gebrauch des Programms gesehen. Therapeuten (30,0 % ±20,92) gingen häufiger als Patienten (19,0 ±23,9) davon aus, dass einige Anwender auf die Unterstützung von Angehörigen oder Therapeuten angewiesen seien.

### Skala Therapeutische Allianz – Revised (STA-R)

**Figure 1:**
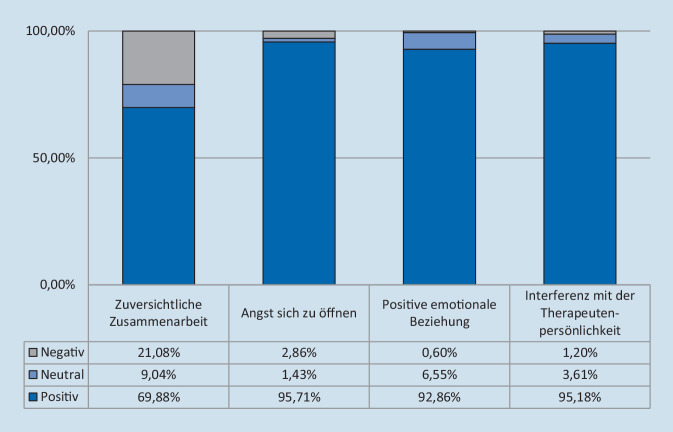

Insgesamt wurde die therapeutische Allianz während der Videotherapie als hoch eingeschätzt. Dabei unterschied sich der Score der Patienten (87,8 % ±9,44) und der Therapeuten (84,8 % ±10,23) nicht signifikant (*p* = 0,14). In den einzelnen Untertests (Abb. 1 und 2) fand sich Folgendes:

**Figure 2:**
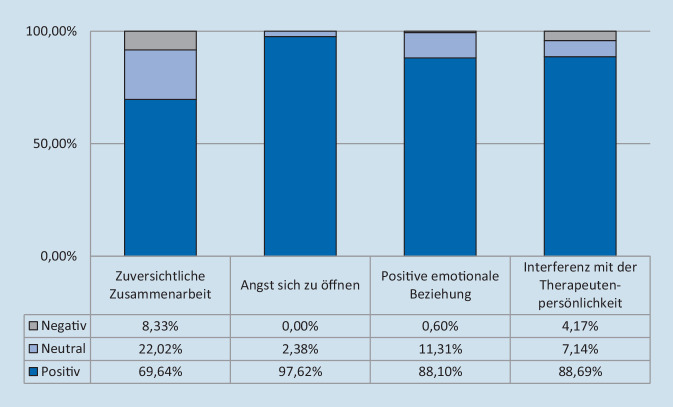

- Die Zuversicht der Patienten hinsichtlich ihrer Zusammenarbeit mit dem Therapeuten (Skala 1) war hoch. In 82,1 % (±27,77) gaben diese an, dass sie ihre Therapieziele durch die Videotherapie erreichen können. Dies galt auch für die Therapeuten (69,01 % ±26,94) (*p* = 0,33).
- Die Auswertung der zweiten Skala zeigte, dass 93,9 % der Patienten keine Angst hatten, sich ihrem Therapeuten zu öffnen. Die Wahrnehmung der Patienten unterschied sich nicht signifikant von der Sichtweise der Therapeuten (*p* = 0,63). Dabei hatte weder das Alter (*p* = 0,75) noch das Geschlecht (*p* = 0,75) einen Einfluss auf die Ängstlichkeit der Patienten, allerdings die Dauer der Hörerfahrung mit dem CI. Je länger die Patienten versorgt waren, desto weniger Angst hatten sie vor der Videotherapie (*p* = 0,0025; τ = 0,325).
- Die dritte Skala, welche die emotionale Beziehung zwischen dem Patienten und dem Therapeuten erfasst, belegte, dass sich die Patienten durch die Videotherapie wertgeschätzt fühlten (94,0 % ±13,31). Dabei zeigte sich ein signifikanter Unterschied zu der Meinung der Therapeuten, die kritischer waren (*p* = 0,00011).
- Im Hinblick auf die Frage einer möglichen Interferenz der Videotherapie mit der Therapeutenpersönlichkeit (Skala 4) unterschied sich die Meinung der Patienten von der ihrer Therapeuten. Während Letztere angaben, dass ihr eigenes Verhalten die Therapie manchmal beeinflusse (81,0 % ±18,51), verneinten alle Patienten dies (100,0 % ±0,0; *p* = 0,0001).

Die Benutzerfreundlichkeit des Programms (SUS-Score) und die therapeutische Allianz waren stark miteinander assoziiert. Je besser die Benutzerfreundlichkeit von den Patienten (*p* = 0,0025; τ = 0,324) und den Therapeuten (*p* = 0,013; τ = 0,265) eingeschätzt wurde, desto stabiler war ihre Beziehung. Darüber hinaus schätzten Therapeuten, die sich sicher in der Anwendung des Programms fühlten (SUS-Score), ihre Patienten als weniger ängstlich ein (STA‑R, Score 2) (*p* = 0,0020; τ = 0,332).

### Kurzfragebogen zur Videotherapie (Tab. 2)

Über den Therapieverlauf zeigten sich zwischen der ersten und fünften Therapieeinheit nur geringfügige Änderungen in der Einschätzung der Therapeuten in Bezug auf die Gesamtqualität der Videotherapie (TE1: 2,56/4 ±0,98; TE5: 3,0/4 ±0,86) sowie deren Umsetzbarkeit (TE1: 2,45/4 ±1,43; TE5: 3,0/4 ±1,22), wobei die Spannweite der Beurteilung durch die einzelnen Therapeuten groß war. Technische Probleme traten in der letzten Sitzung deutlich weniger als zu Beginn auf. Während in der ersten Therapieeinheit vor allem über Schwierigkeiten in der Handhabung, im Umgang mit dem Endgerät, beim Starten des Programms und beim Koppeln des Endgeräts mit der Audioschleife berichtet wurde, zeigten sich diese am Ende der Studie nur noch bei 11,9 % der Sitzungen. Nur die Tonübertragung blieb während des gesamten Studienverlaufs problematisch. Zu einem technisch bedingten Abbruch der Therapieeinheit kam es während der ersten Einheit in vier Fällen (9,5 %). Demgegenüber konnte die 5. Therapieeinheit bei allen Patienten regelrecht durchgeführt werden.

### Abschlussfragebogen zur Videotherapie

In diesem Fragebogen, welcher von Patienten und Therapeuten beantwortet wurde, wurde jeder Bereich separat bewertet (Tab. 4 und 5).

Der Gesamtnutzen der Telemedizin wurde von den Patienten signifikant besser beurteilt als von den Therapeuten (*p* = 0,0065). Patienten mit einer längeren Anreise schätzten die Videotherapie signifikant mehr (*p* = 0,045). Die Qualität des Programms hinsichtlich Verständlichkeit und Bedienbarkeit wurde von Patienten (87,31 % ±17,57) ebenfalls besser bewertet als von den Therapeuten (85,62 % ±16,47). Dieser Unterschied war jedoch nicht signifikant (*p* = 0,49). Eine Ausnahme bildet die Frage, ob das System einfach und leicht zu erlernen sei. Hier antworteten die Therapeuten signifikant positiver (*p* = 0,0033).

Die Qualität der Interaktion erhielt von beiden Usergruppen ein positives Feedback, auch wenn die Patienten etwas kritischer (86,57 % ±15,96) als die Therapeuten (89,26 % ±11,93) waren. Die Zufriedenheit mit dem Konzept der Videotherapie war bei Patienten und Therapeuten ähnlich (*p* = 0,43). Alle Therapeuten (100 %) und 92,86 % der Patienten hielten Videotherapien für eine positive Ergänzung zum Hörtraining vor Ort, und mehr als 80 % der befragten CI-Träger wollten die Videotherapie auch nach der COVID-19-Pandemie weiterhin nutzen.

Die Auswahl und Umsetzung der Therapieinhalte wurden von den Patienten als sehr positiv bewertet (92,33 ±12,79). Je älter die Personen waren, desto besser fiel die Bewertung aus (*p* = 0,019; τ = 0,251).

Auch die Therapeuten gaben an, dass der Einsatz von Text‑, Satz- und Wortmaterial über die Videotherapie problemlos möglich war. Demgegenüber sahen sie die Arbeit auf Silbenebene kritischer. Geräusche wurden kaum eingesetzt (19 %). In 88,0 % konnten die Therapeuten auf bereits vorhandenes Therapiematerial zurückgreifen, um die Videotherapie durchzuführen. Der Einsatz der normalen Sprechstimme gelang in 95 % gut, während dies für eine künstliche Stimme über zusätzliche Computerprogramme nur in 39 % zutraf. Als Hilfestellung wurde das Mundbild häufiger als das Schriftbild verwendet, auch wenn sich Ersteres als störungsanfälliger erwies (29 %).

Die Zufriedenheit mit der Videotherapie korrelierte stark mit der Einschätzung der Beziehung zwischen dem Therapeuten und dem Patienten. Je besser die therapeutische Allianz war, desto zufriedener waren die Patienten (*p* = 0,00064; τ = 0,366) und die Therapeuten (*p* = 0,0020; τ = 0,331) mit der Videotherapie. Außerdem bestand ein größerer subjektiver Nutzen, wenn die therapeutische Allianz positiver eingeschätzt wurde (Patienten: *p* = 0,0042; τ = 0,307; Therapeuten: *p* = 0,029; τ = 0,233). Das Gleiche galt für die Qualität der Interaktion. Je besser die Interaktion bewertet wurde, desto nützlicher wurde die Videotherapie von Patienten (*p* = 0,039; τ = 0,247) und Therapeuten (*p* < 0,000005; τ = 0,568) wahrgenommen.

In den offenen Fragen zu den Vor- und Nachteilen der Videotherapie wurde sowohl von den Patienten (61,9 %) als auch von den Therapeuten (21,4 %) die Zeit- und Kostenersparnis als der größte Vorteil wahrgenommen. Des Weiteren nannten 14,3 % der Patienten und 11,9 % der Therapeuten die verbesserte Trainingseffizienz bei Patienten mit einseitiger Taubheit (SSD), die während der gesamten Therapieeinheit im Unterschied zur Vor-Ort-Therapie über eine Audioschleife trainierten, als Vorteil. Darüber hinaus wurden die Fortführung des Trainings während des Lockdowns (9,5 %) und die flexible Terminvereinbarung (4,8 %) positiv hervorgehoben.

Negativ wurden von den Patienten vor allem technische Probleme bewertet (47,6 %). Des Weiteren gaben 30,9 % der Patienten an, dass der fehlende persönliche Kontakt für sie ein Problem darstellte. Für 7,1 % der Nutzer hatte die Videotherapie keinerlei Nachteil. Fünf Patienten sahen Verbesserungsbedarf hinsichtlich des geteilten Bildschirms. Sie würden den Therapeuten gern auch in dieser Einstellung sehen können. Drei Patienten äußerten den Wunsch nach professionellerer technischer Ausstattung der Patienten und der Therapeuten. Die Möglichkeit der technischen Anpassung würde während der Videotherapie fehlen. Insgesamt 14 Patienten (33,3 %) sahen keinen Verbesserungsbedarf. Für die Therapeuten bestand dieser vor allem im Bereich der Technik (57,1 %). So klagten 34,2 % über Kommunikationsschwierigkeiten aufgrund technischer Probleme. Außerdem fehlten ihnen der persönliche Kontakt und die emotionale Bindung (23,7 %). Des Weiteren erwies sich die Integration von Hintergrundgeräuschen als problematisch.

### Ökonomische Analyse

Wirtschaftliche Vorteile fanden sich vor allem hinsichtlich der Reisekosten. So sparten die Patienten Ø 107,4 (±53,9) min Fahrzeit pro Therapieeinheit. Hochgerechnet auf einen Gesamtrehabilitationsumfang von 40 h Hörtraining im Rahmen der Folgetherapie bei Erwachsenen ergäbe dies eine Einsparung von 76 h. Die Entfernung, die jeder Patient pro Therapiesitzung zurücklegte, betrug Ø 79,5 km (±60,6). Basierend auf einer Fahrtkostenpauschale von 0,30 €/km entfielen für die Patienten Kosten in Höhe von 23,85 €/Therapie (±18,18) und durchschnittlich 954 € für den gesamten Rehabilitationszeitraum. Allerdings benötigten die Therapeuten mehr Zeit für die Vorbereitung (7,5 min/vor Ort vs. 10,5 min/Videotherapie (±5,61)). Dabei hing die Vorbereitungszeit stark vom einzelnen Therapeuten ab. Hierdurch entstanden zusätzliche Kosten von 8,75 €/Patient.

## Diskussion

Wie die vorliegende Studie zeigen konnte, erfährt die Videotherapie eine hohe Akzeptanz bei erwachsenen CI-Trägern und wird auch von Therapeuten gut angenommen, selbst wenn von letztgenannter Seite initial oft Bedenken bestehen [41].

Vor allem die Zeitersparnis wird als Vorteil der Videotherapie gesehen [20, 21]. Dabei treten die ökonomischen Vorteile lediglich auf der Seite der Patienten auf. Für die Rehabilitationseinrichtung dürfte die Videotherapie vor allem in der Anfangszeit, bis digitale Materialien erstellt sind, nicht kosteneffizienter sein. Dies konnte auch Lauer in ihren Experteninterviews an teletherapieerfahrenen Fachtherapeuten aus der Logopädie, Ergotherapie und Physiotherapie belegen [29]. Dabei hängt das Ausmaß einer Kosteneinsparung durch Videotherapien, welche in der Literatur zwischen 12,2 % und 72 % variiert, vor allem von der Entfernung zur Klinik ab [2]. Eine Entfernung von 30 km wird hier als Grenzwert gesehen, ab der die Kosteneinsparung signifikant im Vergleich zur ambulanten Therapie ist [42].

Der Nachteil einer Videotherapie wird seitens der Patienten hauptsächlich im Bereich der Technik gesehen, so in der begrenzten technischen Ausstattung, Problemen in der Handhabung, einer verzögerten Audio- und Videoübertragung oder einer instabilen Internetverbindung [9, 13, 17]. Dies konnte auch durch die vorliegende Studie bestätigt werden, wobei die technischen Probleme mit zunehmender Erfahrung der Patienten abnahmen. Demgegenüber blieb die schwankende Qualität der Audio- und Videoübertragung nahezu unverändert. Dennoch ist davon auszugehen, dass die technischen Probleme in Zukunft mit zunehmender Weiterentwicklung der Digitalisierung lösbar sein werden [29].

Ein in der Literatur kontrovers diskutierter Bereich ist der fehlende persönliche Kontakt zwischen Therapeuten und Patient als Folge des Online-Settings. In der vorliegenden Arbeit vermissten 69 % der Patienten diesen nicht. Diese Beobachtung deckt sich mit einer Studie an 25 Patienten mit depressiven Störungen, Angststörungen oder bipolaren Störungen [9]. Auch wenn eine Videotherapie im Vergleich zu nichtsynchronen Onlineangeboten, wie den zuvor erwähnten Online-Plattformen, einer Vor-Ort-Therapie ähnlicher ist, so stellt diese nach Kühne und Hintenberger keine Face-to-Face-Behandlung, sondern einen „Camera-to-Camera-Kontakt“ dar, der Veränderungen mit sich bringt [28]. So sind Mimik und Gestik der beteiligten Personen durch die Ausschnitthaftigkeit des Videobilds und die Zweidimensionalität nur begrenzt wahrnehmbar und dadurch der direkte Blickkontakt erschwert und Emotionen weniger gut zu erfassen. Des Weiteren sind Unterbrechungen aufgrund technischer Störungen für die Gestaltung einer guten Therapeuten-Patienten-Beziehung hinderlich [18].

Insgesamt beurteilten Patienten, die bereits länger mit einem Cochleaimplantat versorgt waren, die neue Therapieform positiver als solche mit noch geringer Hörerfahrung mit dem CI. Ob dies durch den sichereren Umgang mit dem neuen technischen Gerät, dem besseren Sprachverstehen im Laufe der Rehabilitation oder aber durch den bereits aufgebauten persönlichen Kontakt zum Therapeuten bedingt ist, bleibt offen. Patienten mit einer längeren Anreisedauer schätzen den Nutzen der Videotherapie höher ein [21], solche mit größeren sensorischen, motorischen und kognitiven Problemen haben die größten Schwierigkeiten [16]. Hierzu ist aufgrund der Einschlusskriterien durch die vorliegende Studie keine Aussage möglich.

Allerdings hatte das Alter der Patienten in dieser Studie keinen Einfluss auf die Einschätzung der Benutzerfreundlichkeit, der Zufriedenheit mit dem Medium oder der Qualität der therapeutischen Allianz. Unsere Daten zeigen, dass auch ältere Patienten gut mit dem videotherapeutischen Setting zurechtkommen. Dabei wurden die Therapieinhalte sogar umso besser bewertet, je älter der Patient war (*p* = 0,019). Andere Studien fanden jedoch heraus, dass das Alter, ein unsicherer Umgang mit technischem Equipment und eine instabile psychische Situation Risikofaktoren für das Scheitern von digitalen Angeboten darstellen [5, 11]. Diese unterschiedliche Erfahrung Älterer im Umgang mit digitalen Angeboten liegt womöglich darin begründet, dass die Studienteilnehmer in unserer Studie ausführlich in das Videotool eingewiesen wurden und als Folge der vorangegangenen Vor-Ort-Therapie bereits eine stabile Patienten-Therapeuten-Beziehung bestand. Dies soll entscheidend zum Erfolg einer Online-Therapie beitragen, wie in Studien in der Psychotherapie bereits angemerkt [29, 37].

Demgegenüber stehen Therapeuten einer Videotherapie insgesamt kritischer gegenüber als die Patienten. So soll durch den Einsatz der Videotherapie die emotionale Unterstützung und das Vermitteln von Empathie nach Meinung der Therapeuten weniger gut vermittelt werden und der Kontaktaufbau mit neuen Patienten erschwert sein [7, 40]. Nach Rees bewerten Therapeuten die therapeutische Allianz als signifikant schlechter, wenn sie davon ausgehen, dass der ihnen vorgestellte Therapiemitschnitt aus einer Videotherapie stammt [37]. Möglicherweise ist dies durch das Phänomen des beruflichen Selbstzweifels bedingt, das in der Videotherapie ausgeprägter als im ambulanten Setting sein soll [1].

Ursächlich hierfür könnte die fehlende Sicherheit im Umgang mit Online-Therapie sein. So müssen die Therapeuten sich daran gewöhnen, ihre Verhaltens- und Kommunikationsstrategien an das Online-Setting anzupassen [30, 39]. Daneben ist die konzentrative Belastung höher als durch eine Face-to-Face-Therapie [18]. Um Sprachtherapeuten besser in Theorie, Praxis und Selbsterfahrung für ein qualitätssicherndes Arbeiten im Videosetting zu schulen, erscheint es daher sinnvoll, neue digitale Trainingsformen in das Ausbildungs- und Weiterbildungscurriculum von Therapeuten aufzunehmen [34] und Standards für die Durchführung von Videotherapien zu definieren [18].

Von großer Bedeutung für den Einsatz der Videotherapie in der Hörrehabilitation ist die Bewilligung der Kostenübernahme durch die Kostenträger. Während Telemedizin in Teilen der USA bereits vor der COVID-19-Pandemie komplett finanziert wurde [23], war diese Therapieform vor März 2020 in Deutschland im logopädischen Bereich nicht abrechenbar [15].

Ob eine Videotherapie im Rahmen der Hörrehabilitation ähnlich effektiv wie in einem Face-to-Face-Setting in einem Rehabilitationszentrum ist und dieses phasenweise ergänzen bzw. ersetzen kann, müssen prospektive Studien zeigen. Ein entscheidender Vorteil in der Implementierung teletherapeutischer Konzepte besteht jedoch in einer stärkeren Einbindung des Patienten (Empowerment) [12, 25]. Dies könnte sich langfristig auch positiv auf das Outcome der Rehabilitation auswirken [40].

## Fazit

Unter den evaluierten Gesichtspunkten scheinen Videotherapien nicht nur während der COVID-19-Pandemie eine gute Alternative im Rahmen der Rehabilitation erwachsener CI-Träger darzustellen. Eine erfolgreiche Implementierung setzt jedoch neben einer hohen Motivation, Interesse und Vertrautheit des Therapeuten mit dem neuen Medium auch eine gewisse Technikaffinität des Patienten voraus. Eine stabile therapeutische Allianz, Face-to-Face bereits zuvor aufgebaut, kann hierbei hilfreich sein. Prospektive Langzeitstudien zur Effektivität eines videotherapeutischen Rehabilitationssettings stehen derzeit noch aus und sollten Gegenstand zukünftiger Forschungsprojekte sein.

## Abkürzungen

CI: Cochleaimplantat
SUS: System Usability Scale
STA‑R: Skala Therapeutische Allianz – Revised

## References

[1] Aafjes-van Doorn K, Békés V, Prout TA (2020). Grappling with our therapeutic relationship and professional self-doubt during COVID-19: will we use video therapy again?. Couns Psychol Q.

[2] Aponte-Tinao LA, Farfalli GL, Albergo JI (2019). Face to face appointment vs. telemedicine in first time appointment orthopedic oncology patients: a cost analysis. Stud Health Technol Inform.

[3] Aschendorff A, Arndt S, Kröger S (2020). Qualität der Cochleaimplantat-Rehabilitation unter COVID-19-Bedingungen. Englische Version. HNO.

[4] Ayas M, Al Amadi A, Mohd AH, Khaled D (2020). Impact of COVID-19 on the access to hearing health care services for children with cochlear implants: a survey of parents. F1000Research.

[5] Banducci AN, Weiss NH (2020). Caring for patients with posttraumatic stress and substance use disorders during the COVID-19 pandemic. Psychol Trauma.

[6] Barthel M, Schwinn S, Borgetto B et al. (2021) Digitalisierungschancen –Spurensuche nach Evidenz. Ergebnisse der Videointeraktionsanalyse aus dem Forschungsprojekt „ViTaL“

[7] Berger T (2017). The therapeutic alliance in internet interventions: a narrative review and suggestions for future research. Psychother Res.

[8] Berry K, Salter A, Morris R (2018). Assessing therapeutic alliance in the context of mhealth interventions for mental health problems: development of the mobile agnew relationship measure (mARM) questionnaire. J Med Internet Res.

[9] Brauner L (2015). Acceptability of telepsychiatry in a rural kentucky community mental health clinic.

[10] Brockmann J, Kirsch H, Hatcher R (2011). Dimensionen der therapeutischen Beziehung aus Patienten-Perspektive–Entwicklung der „Skala Therapeutische Allianz-Revised STA-R”. Psychother Psych Med.

[11] Bujnowska-Fedak MM, Grata-Borkowska U (2015). Use of telemedicine-based care for the aging and elderly: promises and pitfalls. SHTT.

[12] Button K, Roos PE, Spasić I (2015). The clinical effectiveness of self-care interventions with an exercise component to manage knee conditions: A systematic review. The Knee.

[13] Davis C, Ng KC, Oh JY (2020). Caring for children and adolescents with eating disorders in the current Coronavirus 19 pandemic: a Singapore perspective. J Adolesc Health.

[14] Deutsche Gesellschaft für Hals-Nasen-Ohren-Heilkunde Kopf- und Hals-Chirurgie e. V. S2k-Leitlinie. https://www.awmf.org/uploads/tx_szleitlinien/017-071l_S2k_Cochlea-Implantat-Versorgung-zentral-auditorische-Implantate_2020-12.pdf. Zugegriffen: 9. Febr. 2021 10.1055/s-0041-1727586 PMC8313172 34154023

[15] Deutscher Bundesverband für Logopädie e. V. Zusatzinformation zur Bestätigung von Videobehandlungen. https://www.dbl-ev.de/service/meldungen/meldung/news/korrigierte-meldung-zu-teletherapievideobehandlung/ (Erstellt: 24. März 2020). Zugegriffen: 9. Febr. 2021

[16] Dial HR, Hinshelwood HA, Grasso SM (2019). Investigating the utility of teletherapy in individuals with primary progressive aphasia. Clin Interv Aging.

[17] Eccleston C, Blyth FM, Dear BF (2020). Managing patients with chronic pain during the COVID-19 outbreak: considerations for the rapid introduction of remotely supported (eHealth) pain management services. Pain.

[18] Eichenberg C (2021). Onlinepsychotherapie in Zeiten der Coronapandemie. Psychotherapeut.

[19] Golinelli D, Boetto E, Carullo G (2020). Adoption of digital technologies in health care during the COVID-19 pandemic: systematic review of early scientific literature. J Med Internet Res.

[20] Gordon CM, Katzman DK (2020). Lessons learned in caring for adolescents with eating disorders: the Singapore experience. J Adolesc Health.

[21] Hagge D, Knopf A, Hofauer B (2020). Chancen und Einsatzmöglichkeiten von Telemedizin in der Hals‑, Nasen- und Ohrenheilkunde bei der Bekämpfung von SARS-COV-2. HNO.

[22] Hall N, Boisvert M, Steele R (2013). Telepractice in the assessment and treatment of individuals with aphasia: a systematic review. Int J Telerehabil.

[23] Hollander JE, Carr BG (2020). Virtually perfect? Telemedicine for Covid-19. N Engl J Med.

[24] Illg A (2017). Rehabilitation bei Kindern und Erwachsenen. HNO.

[25] Jensen CM, Overgaard S, Wiil UK (2019). Can Tele-Health Support Self-Care and Empowerment? A Qualitative Study of Hip Fracture Patients’ Experiences With Testing an “App”.

[26] Kassenärztliche Bundesvereinigung Videosprechstunde: telemedizinisch gestützte Betreuung von Patienten. https://www.kbv.de/html/videosprechstunde.php#:~:text=Seit%201.,werden%2C%20ist%20ebenfalls%20seit%201 (Erstellt: 4. Febr. 2021). Zugegriffen: 9. Febr. 2021

[27] Knaevelsrud C, Maercker A (2007). Internet-based treatment for PTSD reduces distress and facilitates the development of a strong therapeutic alliance: a randomized controlled clinical trial. BMC Psychiatry.

[28] Kühne S, Hintenberger G (2011). Handbuch Online-Beratung.

[29] Lauer N (2020). Teletherapie – hat die Logopädie eine digitale Zukunft?. Forum Logop.

[30] Lawton M, Sage K, Haddock G (2018). Speech and language therapists’ perspectives of therapeutic alliance construction and maintenance in aphasia rehabilitation post-stroke. Int J Lang Commun Disord.

[31] Lewis JR (2018). The system usability scale: past, present, and future. Int J Hum Comput Interact.

[32] Lopez A, Rothberg B, Reaser E (2020). Therapeutic groups via video teleconferencing and the impact on group cohesion. Mhealth.

[33] Mashima PA, Doarn CR (2008). Overview of telehealth activities in speech-language pathology. Telemed J E Health.

[34] Molini-Avejonas DR, Rondon-Melo S, La Amato CAd H (2015). A systematic review of the use of telehealth in speech, language and hearing sciences. J Telemed Telecare.

[35] Nelson E-L, Patton S (2016). Using Videoconferencing to deliver individual therapy and pediatric psychology interventions with children and adolescents. J Child Adolesc Psychopharmacol.

[36] Penedo JMG, Babl AM, Holtforth M (2020). The Association of Therapeutic Alliance with long-term outcome in a guided Internet intervention for depression: secondary analysis from a randomized control trial. J Med Internet Res.

[37] Rees CS, Stone S (2005). Therapeutic alliance in face-to-face versus videoconferenced psychotherapy. Prof Psychol Res Pract.

[38] Reis M, Boisvert I, Beedell E (2019). Auditory training for adult cochlear implant users: a survey and cost analysis study. Ear Hear.

[39] Simpson S, Richardson L, Pietrabissa G (2020). Videotherapy and therapeutic alliance in the age of COVID-19. Clin Psychol Psychother.

[40] Simpson SG, Reid CL (2014). Therapeutic alliance in videoconferencing psychotherapy: a review. The Australian journal of rural health.

[41] Topooco N, Riper H, Araya R (2017). Attitudes towards digital treatment for depression: a European stakeholder survey. Internet Interv.

[42] Tousignant M, Moffet H, Nadeau S (2015). Cost analysis of in-home telerehabilitation for post-knee arthroplasty. J Med Internet Res.

[43] Völter C, Schirmer C, Stöckmann C (2020). Computerbasiertes Hörtraining in der Hörrehabilitation Erwachsener nach Cochleaimplantation. HNO.

[44] Völter C, Schirmer C, Röber M (2021). Neue Wege in der Hörrehabilitation nach Cochleaimplantation. HNO.

[45] Völter C, Stöckmann C, Schirmer C (2021). Tablet-based telerehabilitation versus conventional face-to-face rehabilitation after cochlear implantation: prospective intervention pilot study. JMIR Rehabil Assist Technol.

[46] Weidner K, Lowman J (2020). Telepractice for adult speech-language pathology services: a systematic review. Perspect Asha Sigs.

[47] Wizmann H (2017). Effekte eines strukturierten intensiven Hörtrainings auf die kommunikative Kompetenz von Cochlea Implantat-Trägern.

[48] Zeh R, Baumann U (2015). Stationäre Rehabilitationsmaßnahmen bei erwachsenen CI-Trägern: Ergebnisse in Abhängigkeit von der Dauer der Taubheit, Nutzungsdauer und Alter. HNO.

[49] Hoffmeyer-Zlotnik J, Warner U (2007). How to survey education for cross-national comparisons: the Hoffmeyer-Zlotnik/Warner-Matrix of education. Metodol Zvezki.

